# Supplementary material for: Enhancer and super-enhancer dynamics in repair after ischemic acute kidney injury
Source: Nat Commun. 2020 Jul 7;11:3383. doi: 10.1038/s41467-020-17205-5 (PMC7341735; doi:10.1038/s41467-020-17205-5)
Supplement: Supplementary file 1 — Supplementary Information [file 41467_2020_17205_MOESM1_ESM.pdf]

SUPPLEMENTARY INFORMATION

Enhancer and Super-Enhancer Dynamics in Repair after Ischemic Acute Kidney Injury

Wilflingseder et al.

Supplementary Figure 1-8

Analysis Approach

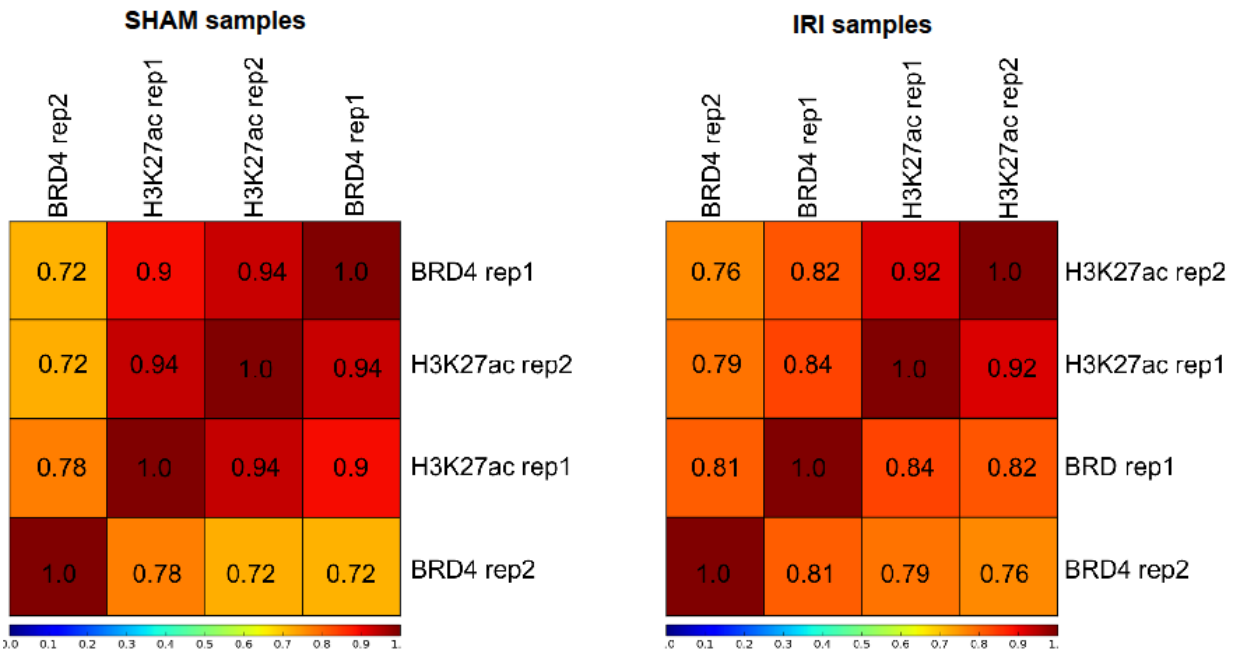

Suppl. Figure 1. Pearson correlation of H3K27ac and BRD4 ChIP-seq peaks in SHAM and IRI samples. H3K27ac and BRD4 are co-localized with high correlations in all replicates between SHAM and IRI samples.

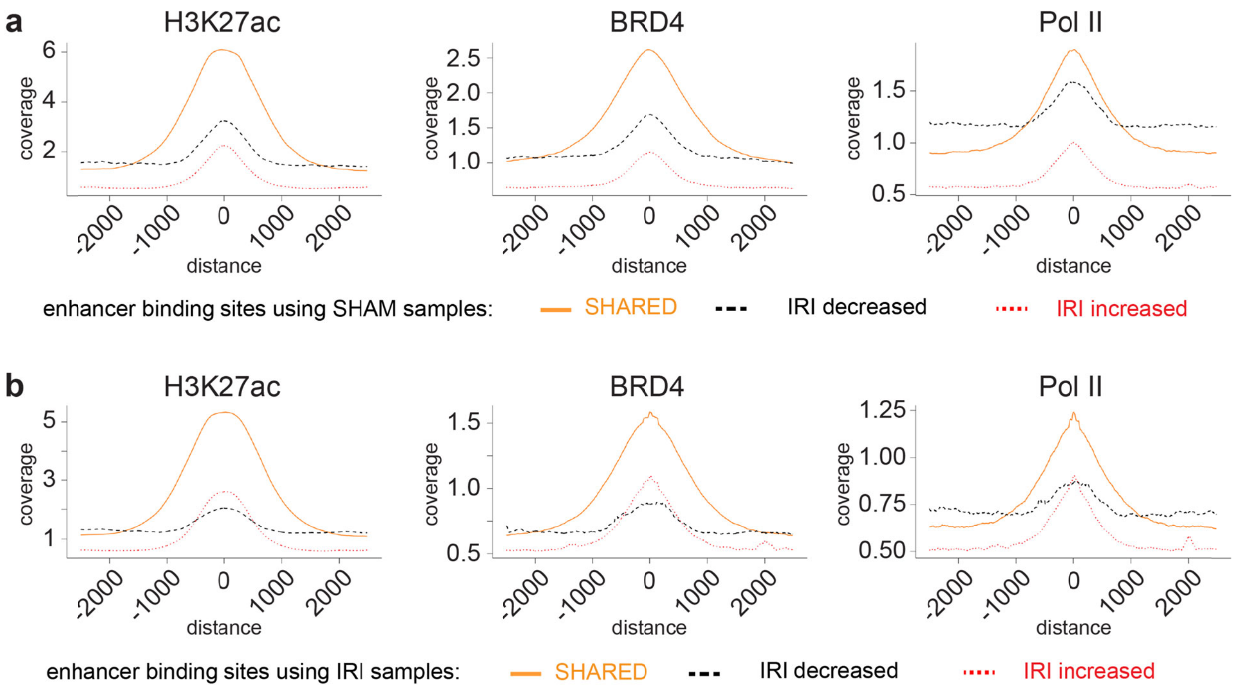

Suppl. Figure 2. Peak coverage of enhancer elements. (a) The coverage plots (using SHAM files) of the enhancer elements show that the peak height of the enhancers identified in SHARED (solid orange line) is for H3K27ac, BRD4 and Pol II the highest, followed by IRI-decreased enhancers (dashed black line) and last by the IRI up-regulated enhancers (dotted red line). **(b)** The coverage plots using the IRI files show that the SHARED enhancers between SHAM and IRI (solid orange line) have the highest coverage in H3K27ac, BRD4 and Pol II. The coverage of the IRI increased enhancers in IRI samples (dotted red line) is slightly higher than the coverage of IRI decreased enhancers with preferential binding in SHAM samples (dashed black line).

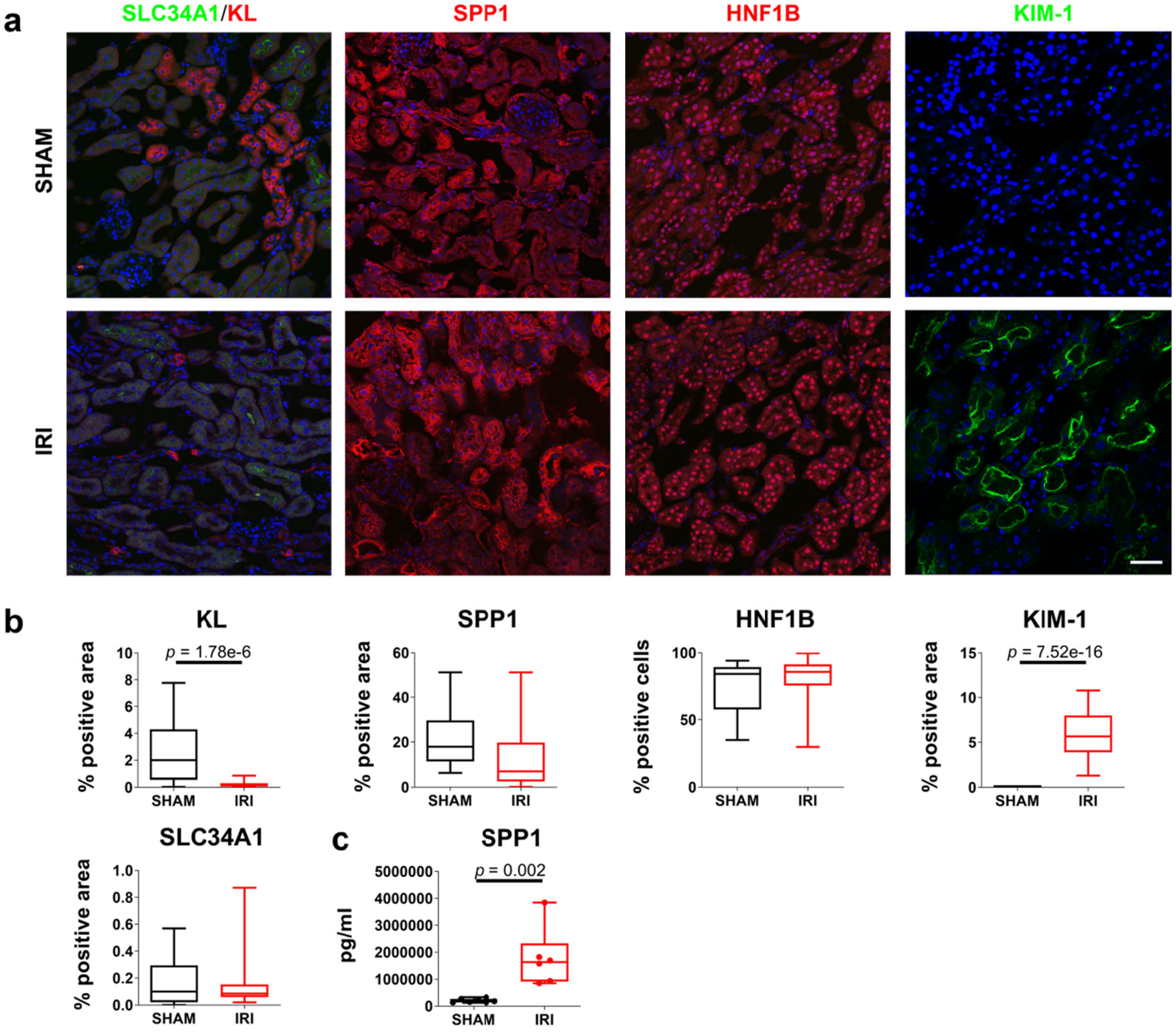

Suppl. Figure 3. Immunofluorescence staining of selected enhancer and super-enhancer associated proteins. (a) Representative immunostaining of SLC34A1/KL, SPP1, HNF1B and KIM-1 in kidney cortex in SHAM (n=4) and IRI (n=6) groups at day 2 after injury and **(b)** quantified percentage of positively stained area (SLC34A1/KL, SPP1, HNF1B, KIM-1) or cells (HNF1B) (at least 4 hpf per sample) **(c)** ELISA of serum SPP1 concentration in SHAM and IRI groups (n=7) at day 2 after injury. t-test (two-sided). Box-plots represents mean ± min, max. Box contains 50% of the data. Scale bar: 50 µm. Source data are provided as a Source Data file.

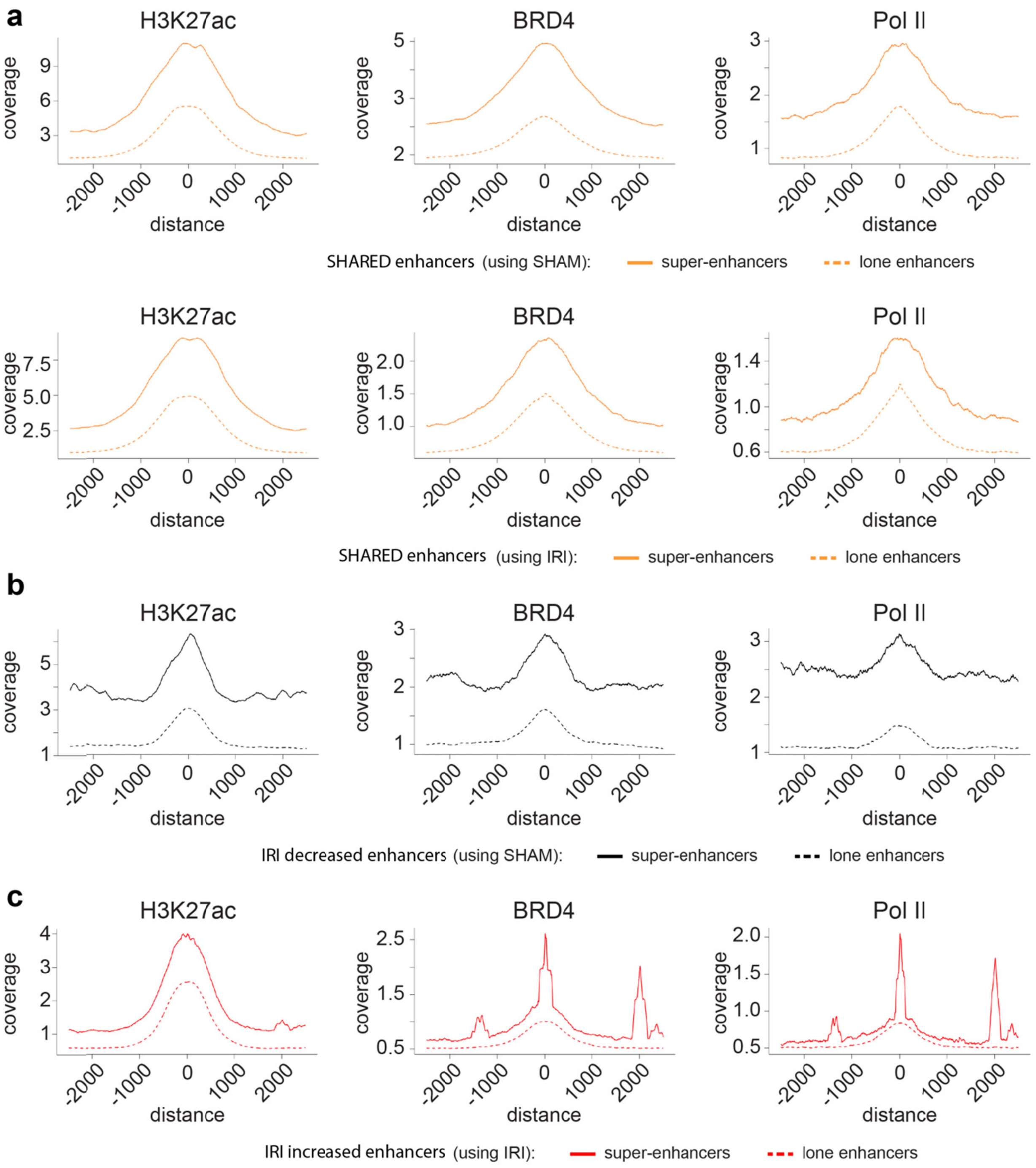

Suppl. Figure 4. Peak coverage of super-enhancers and lone enhancers. (a) Super-enhancers SHARED between SHAM and IRI show a higher peak coverage of H3K27ac, BRD4 and Pol II than SHARED lone enhancers. **(b)** The coverage plots show that IRI decreased super-enhancers have a higher coverage of H3K27ac, BRD4 and Pol II than IRI-decreased lone enhancers. **(c)** H3K27ac, BRD4 and Pol II coverage is higher over IRI-increased super-enhancers than IRI lone enhancers.

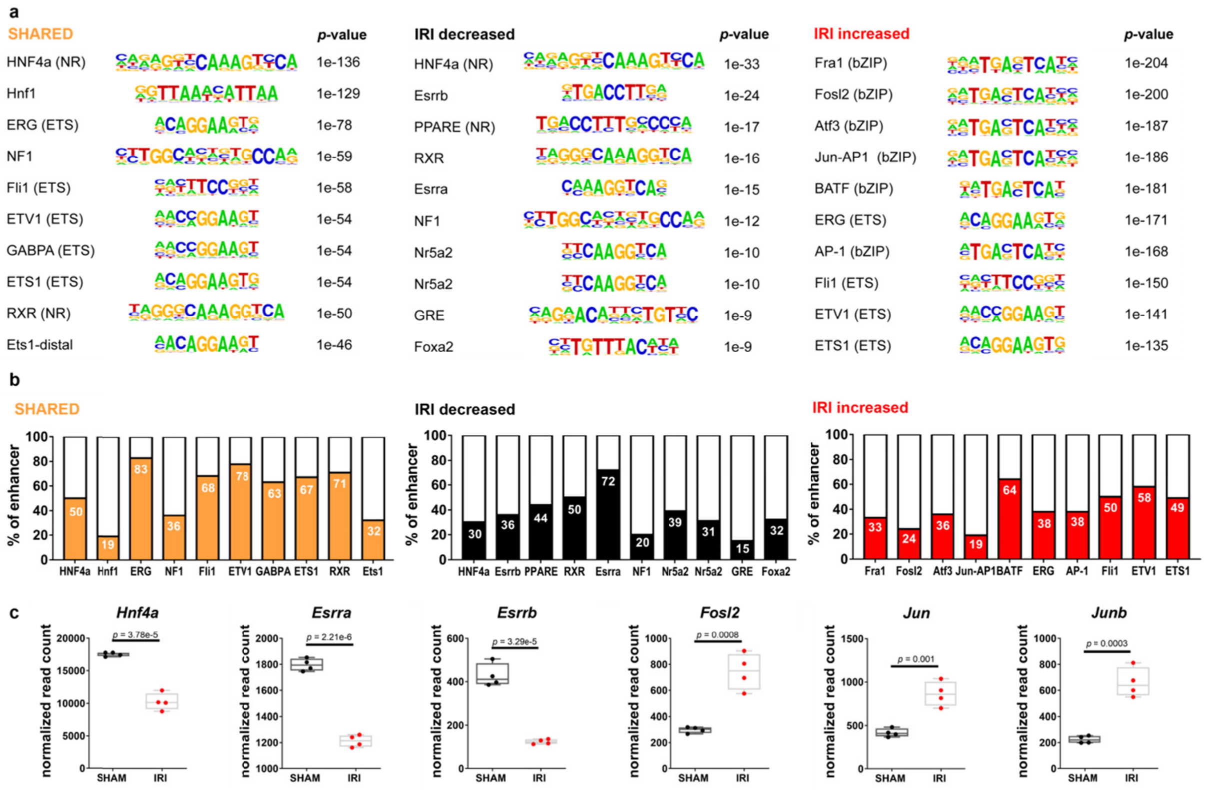

Suppl. Figure 5. Motif analysis of enhancer elements. (a) The top 10 identified transcription factor motifs in each of the three categories, SHARED, IRI-decreased, IRI-increased ranked by p-value. (b) The bar plots show for each category the percentage of enhancers having the specific motif **(c)** mRNA expression levels between SHAM and IRI for representative transcription factors associated with highly enriched binding motifs. n=4; Individual data points and box-plot with mean ± max, min are shown. Two sample t-test was applied (two-sided).

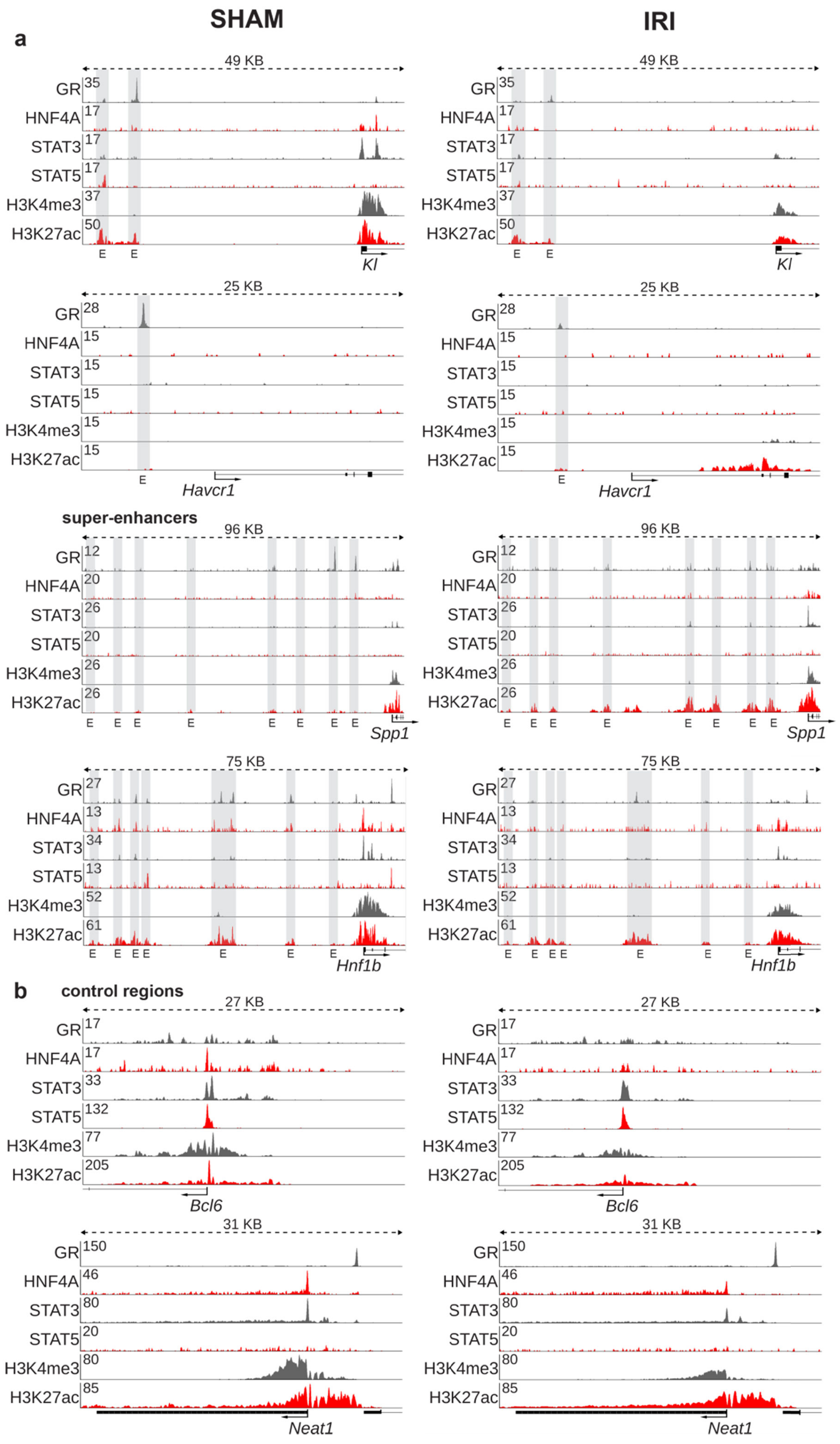

Suppl. Figure 6. Representative examples of transcription factor binding at super-enhancer sites in kidney epithelia cells. (a) The Kl, Havcr1, Spp1 and Hnf1b genomic locus are shown for HNF4A, GR, STAT3 and STAT5 binding together with H3K4me3 and H3K27ac in the SHAM (left) and IRI (right) condition. **(b)** Representative control regions (Bcl6 and Neat1) are shown for transcription factor ChIP-seq quality between SHAM and IRI. Enhancer elements are indicated by grey bars.

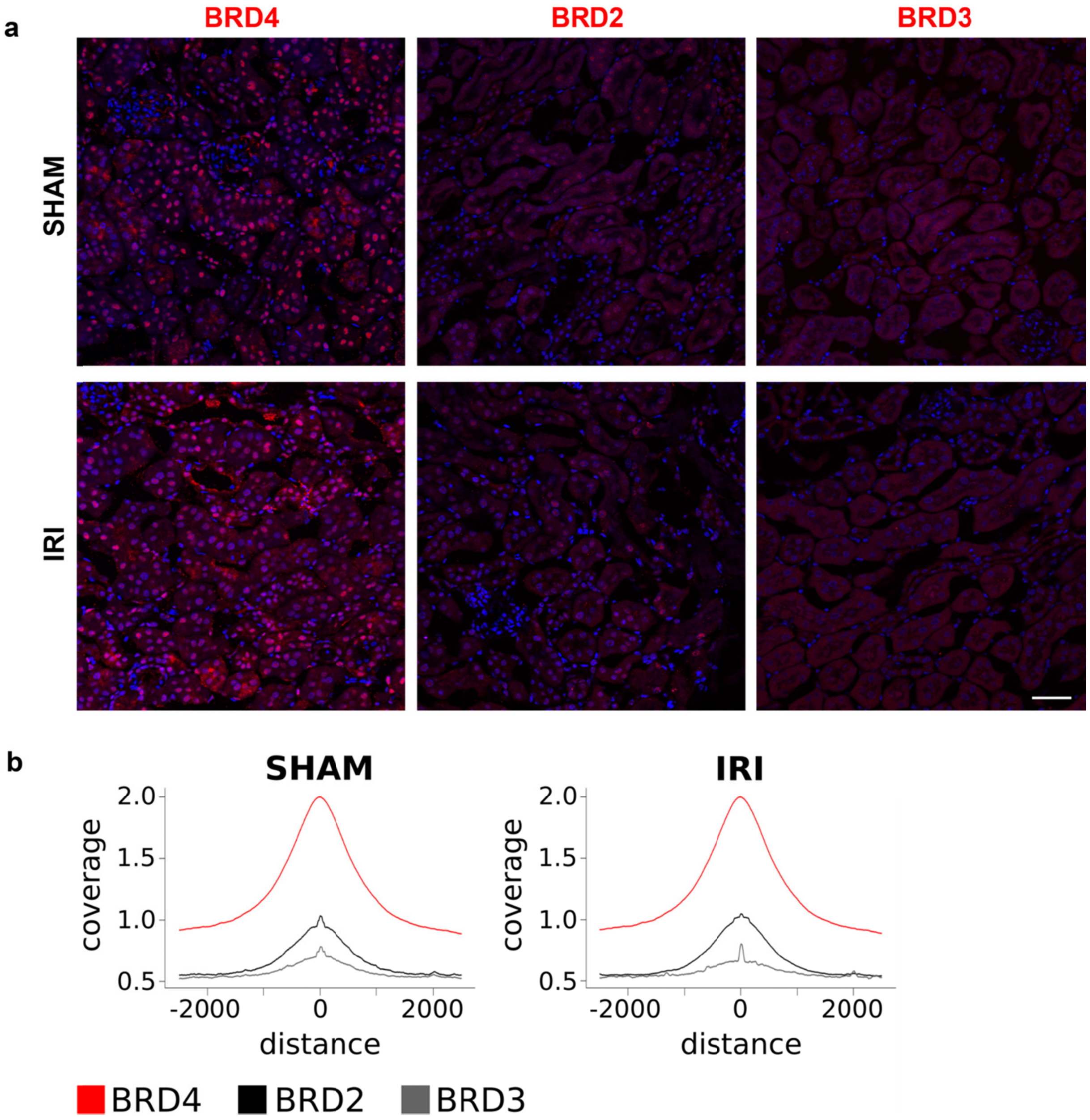

Suppl. Figure 7. Assessment of BET family members: BRD4, BRD2 and BRD3. (a) Representative immunostaining of BRD4, BRD2 and BRD3 in kidney cortex in SHAM and IRI samples at day 2 after injury (Scale bar: 50 μm). Four independent mice in each group were assessed. **(b)** Genome-wide coverage blots of BRD4, BRD2 and BRD3 ChIP-seq profiles in SHAM and IRI. BRD4 is the dominate member of the BET family in kidney cortex.

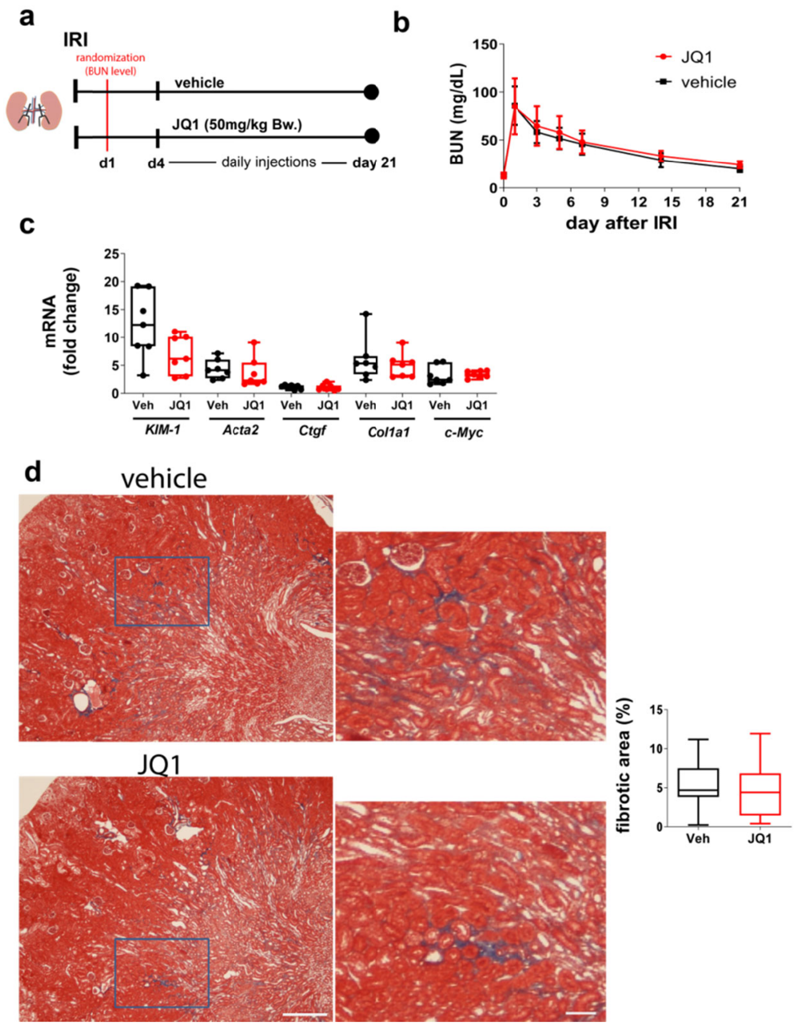

Suppl. Figure 8. **Phenotypic consequences of JQ1 treatment starting at day 4 after IRI. (a)** Male mice on a C57BL/6N background (10- to 12-week-old) were treated daily starting at the day 4 after IRI surgery with JQ1 (50 mg/kg) or vehicle (DMSO / 10% ß-cyclo dextrin 1:10). Mice were assigned to JQ1 or vehicle group by randomization startified by BUN level at day 1. Mice were sacrificed at day 21. **(b)** BUN (mg/dL) trajectories between vehicle and JQ1 treated mice from day 0 until day 21 after injury (mean ± SD). n=7 biologically independent samples **(c)** Fold change for KIM-1 (*Havcr1*), α-SMA (*Acta2*), *Ctgf*, *Col1a1* and *Myc* comparing vehicle and JQ1 treated animals. Individual data points are shown. Box-plot represents the mean ± min, max. The box contains 50% of the data.n=7 biologically independent samples **(d)** Representative trichrome-staining and quantification of fibrotic area of vehicle and JQ1-treated kidneys. Box-plot represents the mean ± min, max. The box contains 50% of the data. Scale bar: 500 µm and 100µm; n=7 (5 hpf per sample); Source data are provided as a Source Data file.

Analysis of sequencing data (ChIP-seq and RNA-seq)

ChIP-seq analysis

1) **Quality control of the samples using fastqc (version 0.11.6)**

```
fastqc -o $QCOUT -a adapters.txt $FASTQF
```

2) **Trimming of the samples using trimmomatic (version 0.36)**

```
java -jar trimmomatic.jar \
   SE \
   $FASTQF \
   $FASTQF_TRIMMING \
   ILLUMINACLIP:TruSeq3-SE.fa:2:30:12 \
   LEADING:20 \
   HEADCROP=HEADCROP:15 \
   TRAILING=TRAILING:20 \
   SLIDINGWINDOW=SLIDINGWINDOW:4:20 \
   MINLEN=MINLEN:20
```

3) **Quality control of the samples after trimming using fastqc (version 0.11.6)**

```
fastqc -o $QCOUT -a adapters.txt $FASTQF_TRIMMING
```

4) **Bowtie and samtools for mapping (bowtie version 1.1.2; samtools 1.3.1)**

```
cd $WD \
&& export BOWTIE_INDEXES=BowtieIndex/ \
&& zcat $FASTQF_TRIMMING \
| bowtie -m 1 --sam genome - \
| samtools view -F4 -Sb -> $MAPPINGOUT
```

5) **Homer for generating bedgraph files (version 4.8.2)**

```
makeTagDirectory $SAMPLE $MAPPINGOUT

makeUCSCfile $SAMPLE -o auto
```

6) **Broad peak calling using MACS2 (version 2.1.1)**

   a. **Samples: SHAM H3K27ac, SHAM H3K4me3, IRI H3K4me3:**

```
macs2 callpeak -t $SAMPLE -f BAM -g mm -B --broad \
--broad-cutoff 0.1
```

b. **Samples: IRI H3K27ac:**

```
macs2 callpeak -t $SAMPLE -f BAM -g mm -B --broad \
--broad-cutoff 0.05
```

7) **Merging peaks of replicates (BEDtools version 2.26.0)**

```
bedtools intersect -wa -a $REPLICATE_1 -b $REPLICATE_2
```

8) **Application of BEDtools (version 2.26.0) to retrieve peaks present in:**

a. **SHAM and IRI**

```
bedtools intersect -wa -a $SHAM_H3K27ac -b $IRI_H3K27ac \
> $preliminary-SHAM-confirmed-peaks.bed
```

b. **IRI and SHAM**

```
bedtools intersect -wa -a $IRI_H3K27ac -b $SHAM_H3K27ac \
> $preliminary-IRI-confirmed-peaks.bed
```

c. **merge peaks present in SHAM and IRI**

```
cat $preliminary-SHAM-confirmed-peaks.bed \
$preliminary-IRI-confirmed-peaks.bed \
> $SHARED-SHAM-IRI-peaks.bed
```

d. **get only SHAM peaks (IRI decreased)**

```
bedtools intersect -wa -v -a $SHAM_H3K27ac -b $IRI_H3K27ac \
> $only-SHAM-peaks.bed
```

e. **get only IRI peaks (IRI increased)**

```
bedtools intersect -wa -v -a $IRI_H3K27ac -b $SHAM_H3K27ac \
> $only-IRI-peaks.bed
```

9) **Categorize peaks as enhancers or promoters**

→ Mouse-TSS.bed was created by downloading the mm10 TSS sites from UCSC genome browser and as subsequent adding of +/-2500bp to the promoter coordinates to define the promoter regions

```
bedtools intersect -wa -a $PEAKFILE_STEP8 -b Mouse-TSS.bed | \
sort |uniq > $PROMOTERS

bedtools intersect -wa -v -a $PEAKFILE_STEP8 -b Mouse-TSS.bed | \
sort |uniq > $ENHANCERS
```

10) **Identify promoters with H3K4me3**

→ overlap identified promoter elements with H3K4me3 peak calling results

```
bedtools intersect -wa -a $PROMOTERS -b $PEAK_CALLING_H3K4me3
```

11) Motif analysis and identification of peaks with underlying motifs

```
findMotifsGenome.pl $ENHANCERS mm10 output -len 8,10,12 -nomotif

annotatePeaks.pl $ENHANCERS mm10 -size given -m known1.motif
```

12) Super-enhancer analysis

```
python ROSE_main.py -g mm10 -i $ENHANCERS.gff -r $BAM -t 2500
```

→ Overlap super-enhancers from H3K27ac and BRD4 using `R` with the package `dplyr`

13) Assign enhancers and super-enhancers to genes

Using GREAT (http://great.stanford.edu/public/html/) with option "Basal plus extension": plus Distal: 50KB, Proximal 5KB; 1KB downstream

14) Categorize enhancers as lone enhancers or elements of super-enhancers

```
bedtools intersect -wa -a $ENHANCERS -b $SUPER_ENHANCERS >
$ENHANCERS_WITHIN_SE

bedtools intersect -wa -v a $ENHANCERS -b $SUPER_ENHANCERS >
$LONE_ENHANCERS
```

15) Peak and super-enhancer coverage was analyzed using Homer

```
annotatePeaks.pl $ENHANCERS mm10 -size 5000 -hist 5 \
-d $BAM_FILE_1 $BAM_FILE_1
```

RNA-seq analysis

1) Quality control of the samples (version 0.11.6)

```
fastqc -o $QCOUT -a adapters.txt $FASTQF
```

2) Trimming of the samples using trimmomatic (version 0.36)

```
java -jar trimmomatic.jar \
SE \
$FASTQF \
$FASTQF_TRIMMING \
ILLUMINACLIP:TruSeq3-SE.fa:2:30:12 \
LEADING:20 \
HEADCROP=HEADCROP:15 \
TRAILING=TRAILING:20 \
SLIDINGWINDOW=SLIDINGWINDOW:4:20 \
MINLEN=MINLEN:20
```

3) **Quality control of the samples after trimming (version 0.11.6)**

```
fastqc -o $QCOUT -a adapters.txt $FASTQF_TRIMMING
```

4) **Mapping using STAR (STAR version 2.5.3a; samtools version 1.3.1)**

```
cd $WP2 \
   && STAR \
   --genomeDir genes-50 \
   --sjdbOverhang 50 \
   --readFilesIn $INPUT \
   --outSAMtype BAM SortedByCoordinate \
   --outFilterMultimapNmax 20 \
   --outReadsUnmapped Fastx \
   --outFileNamePrefix $MAPPING_OUT

samtools sort $MAPPING_OUT -o $MAPPING_OUT_SORT
```

5) **HTSeq for getting gene counts (version 0.6.1p1)**

```
htseq-count -i gene_id -r pos -t exon -f bam -s no
$MAPPING_OUT_SORT $GTF_FILE
```

6) **DESeq2 analysis for differential gene expression**

→ DESeq2 was used for differential gene expression

Integration of ChIP-seq and RNA-seq data

Combine enhancers with RNA-seq data by matching the gene names of both analyses in `R` using the package `dplyr`.

Data availability

All sequencing data, raw files as well as processed data (bedGraph and gene counts) are available in the Gene Expression Omnibus (GEO) at NCBI with the accession number GSE114294.

To review GEO accession GSE114294:
Go to https://www.ncbi.nlm.nih.gov/geo/query/acc.cgi?acc=GSE114294
Enter token ehmhuyygtfoblyl into the box

SUPPLEMENTARY REFERENCES

1. Love MI, Huber W, Anders S. Moderated estimation of fold change and dispersion for RNA-seq data with DESeq2. *Genome Biol* **15**, 550 (2014).

2. McLean CY*, et al.* GREAT improves functional interpretation of cis-regulatory regions. *Nat Biotechnol* **28**, 495-501 (2010).

3. Heinz S*, et al.* Simple combinations of lineage-determining transcription factors prime cis-regulatory elements required for macrophage and B cell identities. *Mol Cell* **38**, 576-589 (2010).

4. Uhlén M*, et al.* Proteomics. Tissue-based map of the human proteome. *Science* **347**, 1260419 (2015).
