# Supplementary material for: Enhancer and super-enhancer dynamics in repair after ischemic acute kidney injury
Source: Nat Commun. 2020 Jul 7;11:3383. doi: 10.1038/s41467-020-17205-5 (PMC7341735; doi:10.1038/s41467-020-17205-5)
Supplement: Supplementary file 3 — Description of Additional Supplementary Information [file 41467_2020_17205_MOESM3_ESM.pdf]

Supplementary Data 1 - 1716 up-regulated genes in IRI samples on day 2 (n=4) compared to SHAM (n=4) with false discovery rate (FDR) < 5% and fold change +/- 2. RNA-seq differential gene expression was done using DESeq2 [1]. The function of the R package DESeq2 (Differential expression analysis based on Negative Binomial distribution) is to perform three steps: (i) estimation of size factors; (ii) estimation of dispersion; (iii) Negative Binomial GLM fitting and Wald statistics[1].

Supplementary Data 2 - 919 down-regulated genes in IRI samples on day 2 (n=4) compared to SHAM (n=4). RNA-seq differential gene expression was determined using DESeq2 [1]. The function of the R package DESeq2 (Differential expression analysis based on Negative Binomial distribution) is to perform three steps: (i) estimation of size factors; (ii) estimation of dispersion; (iii) Negative Binomial GLM fitting and Wald statistics[1].

Supplementary Data 3 – SHARED enhancer - gene assignment. Enhancers were assigned to the 'nearest' gene (within 100kb) using GREAT[2].

Supplementary Data 4 – IRI decreased enhancer - gene assignment. Enhancers were assigned to the 'nearest' gene (within 100kb to the TTS) using GREAT[2].

Supplementary Data 5 – IRI increased enhancer - gene assignment. Enhancers were assigned to the 'nearest' gene (within 100kb to the TSS) using GREAT[2].

Supplementary Data 6 – IRI decreased super-enhancer - gene assignment. Super-enhancers were assigned to the 'nearest' gene (within 100kb to the TTS) using GREAT[2].

Supplementary Data 7 – IRI increased super-enhancer - gene assignment. Super-enhancers were assigned to the 'nearest' gene (within 100kb to the TTS) using GREAT[2].

Supplementary Data 8 – SHARED super-enhancer - gene assignment. Super-enhancers were assigned to the 'nearest' gene (within 100kb to the TTS) using GREAT[2].

Supplementary Data 9 - All identified transcription factor motifs in each of the three categories, SHARED, IRI-decreased, IRI-increased ranked by p-value. HOMER[3] was used for motif analysis using the default settings.

Supplementary Data 10 - 676 up-regulated genes in the IRI JQ1 day 2 kidneys (n=4) compared to IRI day 2 kidneys (n=4) with false discovery rate (FDR) < 5% and fold change +/- 2. RNA-seq differential gene expression was done using DESeq2 [1]. The function of the R package DESeq2 (Differential expression analysis based on Negative Binomial distribution) is to perform three steps: (i) estimation of size factors; (ii) estimation of dispersion; (iii) Negative Binomial GLM fitting and Wald statistics[1].

Supplementary Data 11 - 2378 down-regulated genes in IRI JQ1 day 2 kidneys (n=4) compared to IRI day 2 kidneys (n=4) with false discovery rate (FDR) < 5% and fold change +/- 2. RNA-seq differential gene expression was done using DESeq2 [1]. The function of the R package DESeq2 (Differential expression analysis based on Negative Binomial distribution) is to perform three steps: (i) estimation of size factors; (ii) estimation of dispersion; (iii) Negative Binomial GLM fitting and Wald statistics[1].

Supplementary Data 12 - 774 up-regulated genes in SHAM JQ1 day 2 kidneys (n=3) compared to SHAM vehicle day 2 kidneys (n=4) with false discovery rate (FDR) < 5% and fold change +/- 2. RNA-seq differential gene expression was done using DESeq2 [1]. The function of the R package DESeq2 (Differential expression analysis based on Negative Binomial distribution) is to perform three steps: (i) estimation of size factors; (ii) estimation of dispersion; (iii) Negative Binomial GLM fitting and Wald statistics[1].

Supplementary Data 13 - 1667 down-regulated genes in SHAM JQ1 day 2 kidneys (n=3) compared to SHAM vehicle day 2 kidneys (n=4) with false discovery rate (FDR) < 5% and fold change +/- 2. RNA-seq differential gene expression was done using DESeq2 [1]. The function of the R package DESeq2 (Differential expression analysis based on Negative Binomial distribution) is to perform three steps: (i) estimation of size factors; (ii) estimation of dispersion; (iii) Negative Binomial GLM fitting and Wald statistics[1].

Supplementary Data 14 - Gene Ontology (GO) term: molecular function significantly enriched by down-regulated genes (1667 genes) after two days of JQ1 treatment in SHAM kidneys.

Supplementary Data 15 – List of genes elevated in kidney tissue based on RNA-seq analysis comparing human tissues [4]. Marked in light blue: genes also down-regulated under JQ1 treatment in SHAM kidneys
