# Supplementary material for: Enhancer and super-enhancer dynamics in repair after ischemic acute kidney injury
Source: Nat Commun. 2020 Jul 7;11:3383. doi: 10.1038/s41467-020-17205-5 (PMC7341735; doi:10.1038/s41467-020-17205-5)
Supplement: Supplementary file 19 — Reporting summary [file 41467_2020_17205_MOESM19_ESM.pdf]

nature research

Corresponding author(s): Joseph V. Bonventre
Julia Wilflingseder

Last updated by author(s): Jun 2, 2020

Reporting Summary

Nature Research wishes to improve the reproducibility of the work that we publish. This form provides structure for consistency and transparency in reporting. For further information on Nature Research policies, seeAuthors & Referees and theEditorial Policy Checklist .

Statistics

For all statistical analyses, confirm that the following items are present in the figure legend, table legend, main text, or Methods section.

n/a	Confirmed	
☐	☒	The exact sample size (*n*) for each experimental group/condition, given as a discrete number and unit of measurement
☐	☒	A statement on whether measurements were taken from distinct samples or whether the same sample was measured repeatedly
☐	☒	The statistical test(s) used AND whether they are one- or two-sided<br>*Only common tests should be described solely by name; describe more complex techniques in the Methods section.*
☐	☒	A description of all covariates tested
☐	☒	A description of any assumptions or corrections, such as tests of normality and adjustment for multiple comparisons
☐	☒	A full description of the statistical parameters including central tendency (e.g. means) or other basic estimates (e.g. regression coefficient) AND variation (e.g. standard deviation) or associated estimates of uncertainty (e.g. confidence intervals)
☐	☒	For null hypothesis testing, the test statistic (e.g. *F*, *t*, *r*) with confidence intervals, effect sizes, degrees of freedom and *P* value noted<br>*Give P values as exact values whenever suitable.*
☒	☐	For Bayesian analysis, information on the choice of priors and Markov chain Monte Carlo settings
☒	☐	For hierarchical and complex designs, identification of the appropriate level for tests and full reporting of outcomes
☐	☒	Estimates of effect sizes (e.g. Cohen's *d*, Pearson's *r*), indicating how they were calculated

Our web collection on statistics for biologists contains articles on many of the points above.

Software and code

Policy information about availability of computer code

Data collection	No software were used for data collection.
Data analysis	We only applied existing tools (listed below) to analyze our data, but did not develop any new software or code, which is not already published.<br>fastqc (v 0.11.6); trimmomatic (v 0.36); bowtie (v 1.1.2); samtools (v 1.3.1); homer (v 4.8.2); MACS2 (v 2.1.1); BEDtools (v 2.26.0); ROSE (super-enhancer) algorithm; GREAT; STAR (v 2.5.3a); HTSeq (v 0.6.1p1); DESeq2; R with the packages dplyr and ggplot2; Image J (bundled with 64-bit Java 1.8.0)

For manuscripts utilizing custom algorithms or software that are central to the research but not yet described in published literature, software must be made available to editors/reviewers. We strongly encourage code deposition in a community repository (e.g. GitHub). See the Nature Research guidelines for submitting code & software for further information.

Data

Policy information about availability of data

All manuscripts must include a data availability statement. This statement should provide the following information, where applicable:

- Accession codes, unique identifiers, or web links for publicly available datasets
- A list of figures that have associated raw data
- A description of any restrictions on data availability

ChIP-seq and RNA-seq raw data files are available in the Gene Expression Omnibus (GEO) at NCBI with the accession number GSE114294.
Figure 1-4 and 6
The source data underlying Figs 5b and e, 6b, g and h, 7 b, e and h and Supplementary Figs S3 b-c and S8 b-d are provided as a Source Data file.
no restrictions on data availablility

Field-specific reporting

Please select the one below that is the best fit for your research. If you are not sure, read the appropriate sections before making your selection.

[x] Life sciences [ ] Behavioural & social sciences [ ] Ecological, evolutionary & environmental sciences

For a reference copy of the document with all sections, see nature.com/documents/nr-reporting-summary-flat.pdf

Life sciences study design

All studies must disclose on these points even when the disclosure is negative.

Sample size	no sample size calculation was performed
Data exclusions	no data were excluded
Replication	no replication was performed
Randomization	animals were randomly assigned to experimental groups. In one experiment we stratified the animals based on BUN levels at day 1 after ischemia reperfusion injury (IRI).
Blinding	Only the surgeon who was conducting the IRI and unilateral ureter obstruction (UUO) surgery was blinded to treatment (JQ1 or vehicle). For all other experiments investigators were not blinded.

Reporting for specific materials, systems and methods

We require information from authors about some types of materials, experimental systems and methods used in many studies. Here, indicate whether each material, system or method listed is relevant to your study. If you are not sure if a list item applies to your research, read the appropriate section before selecting a response.

Materials & experimental systems

n/a	Involved in the study	
[ ]	[x]	Antibodies
[x]	[ ]	Eukaryotic cell lines
[x]	[ ]	Palaeontology
[ ]	[x]	Animals and other organisms
[x]	[ ]	Human research participants
[x]	[ ]	Clinical data

Methods

n/a	Involved in the study	
[ ]	[x]	ChIP-seq
[x]	[ ]	Flow cytometry
[x]	[ ]	MRI-based neuroimaging

Antibodies

Antibodies used

For ChIP-seq: anti-H3K27ac (Abcam, ab4729), anti-H3K4me3 (Millipore, 17-614), anti-BRD4 (Behtyl Laboratory, A301-985A), and anti-RNA Polymerase II (Abcam, ab5408), anti-BRD2 (Bethyl Laboratory, A301-583A), anti-BRD3 (Bethyl Laboratory, A301-368A), anti- HNF4A (Abcam, ab41898), anti-GR (Thermo Scientific, PA1-511A), anti-STAT3 (Santa Cruz, sc-482) and anti-STAT5 (Santa Cruz, sc-835);

IF staining: rabbit antibody to Ki-67 (Vector - VP-K451, 1 in 200), rabbit antibody to KIM-1 (LSBio, R9, 1 in 200), rabbit antibody to α-SMA (Sigma, A5228, 1 in 400), rabbit anti-Hnf1b (Thermo Fisher Scientific, 720259, 1 in 200), rabbit anti-GR (Thermo Fisher Scientific, PA1-511A, 1 in 200), rabbit anti-Slc34a1 (Novusbio, NBP2-13328, 1 in 200), rabbit anti-Spp1 (Abcam, ab8448) and rat anti-Kl (BioLogo, KM2076, 1 in 200), rat anti F4/80 (Abcam, ab6640, 1 in 1000), FITC abeled secondary antibody (Jackson ImmunoResearch, Code: 111-095-003), Cy3 abeled secondary antibody (Jackson ImmunoResearch, Code: 109-165-003)

Validation

anti-H3K27ac, anti-H3K4me3, anti-RNA Polymerase II, anti-GR, anti-STAT3 and anti-STAT5 primary antibodies for ChIP-seq were used in following publication: Shin HY, Willi M, HyunYoo K, et al. Hierarchy within the mammary STAT5-driven Wap super-enhancer. Nat Genet. 2016;48(8):904-911. doi:10.1038/ng.3606

anti-BRD4, anti-BRD2 and anti-BRD3 primary antibodies for ChIP-seq were used in following publications: Lovén J, Hoke HA, Lin CY, et al. Selective inhibition of tumor oncogenes by disruption of super-enhancers. Cell. 2013;153(2):320-334. doi:10.1016/j.cell.2013.03.036; Xu L, Chen Y, Mayakonda A, et al. Targetable BET proteins- and E2F1-dependent transcriptional program maintains the malignancy of glioblastoma. Proc Natl Acad Sci U S A. 2018;115(22):E5086-E5095. doi:10.1073/pnas.1712363115

anti-HNF4A primary ntibodies for ChIP-seq were used in following publication: Qu M, Duffy T, Hirota T, Kay SA. Nuclear receptor HNF4A transrepresses CLOCK:BMAL1 and modulates tissue-specific circadian networks. Proc Natl Acad Sci U S A. 2018;115(52):E12305-E12312. doi:10.1073/pnas.1816411115

primary antibodies for IF staining were used in following publications: Yang L, Besschetnova TY, Brooks CR, Shah JV, Bonventre JV. Epithelial cell cycle arrest in G2/M mediates kidney fibrosis after injury. Nat Med. 2010;16(5):535-143. doi:10.1038/nm.2144; Lemos DR, McMurdo M, Karaca G, et al. Interleukin-1β Activates a MYC-Dependent Metabolic Switch in Kidney Stromal Cells

Necessary for Progressive Tubulointerstitial Fibrosis. J Am Soc Nephrol. 2018;29(6):1690-1705. doi:10.1681/ASN.2017121283; Kishi S, Brooks CR, Taguchi K, et al. Proximal tubule ATR regulates DNA repair to prevent maladaptive renal injury responses. J Clin Invest. 2019;129(11):4797-4816. doi:10.1172/JCI122313; Lindberg K, Amin R, Moe OW, et al. The kidney is the principal organ mediating klotho effects. J Am Soc Nephrol. 2014;25(10):2169-2175. doi:10.1681/ASN.2013111209

Animals and other organisms

Policy information about studies involving animals; ARRIVE guidelines recommended for reporting animal research

Laboratory animals	Male C57BL/6N or BALB/c mice aged 8 to 10 weeks weighing 20–22 g were purchased from Charles River Laboratories. At start of the experiments the mice were 10 to 12 weeks old. Housing conditions: 22-24°C ambient temperature, 50-60% humidity, 12 hours dark/light cycle
Wild animals	no wild animals were used
Field-collected samples	no field-collected samples were used
Ethics oversight	All mouse work was performed in accordance with the animal use protocol approved by the Institutional Animal Care and User Committee of the Harvard Medical School / Brigham and Women's Hospital

Note that full information on the approval of the study protocol must also be provided in the manuscript.

ChIP-seq

Data deposition

- [x] Confirm that both raw and final processed data have been deposited in a public database such as GEO.
- [x] Confirm that you have deposited or provided access to graph files (e.g. BED files) for the called peaks.

Data access links<br>*May remain private before publication.*	GEO accession GSE114294:<br>Go to https://www.ncbi.nlm.nih.gov/geo/query/acc.cgi?acc=GSE114294
Files in database submission	ChIP-seq Sham and IRI (2 replicates each): Brd4, H3K27ac, Pol-II, H3K4me3, Brd2, Brd3, Hnf4a, GR, STAT3 and STAT5;
Genome browser session<br>(e.g. UCSC)	all raw (fastq) and processed (bedGraph) data are available in GEO

Methodology

Replicates	Two replicates per antibody and condition
Sequencing depth	ChIP-seq: Single end and 50bp<br>ChIP-seq raw read counts: between 23.557.618 and 56.558.408
Antibodies	anti-H3K27ac (Abcam, ab4729), anti-H3K4me3 (Millipore, 17-614), anti-BRD4 (Behtyl Laboratory, A301-985A), and anti-RNA Polymerase II (Abcam, ab5408), anti-BRD2 (Bethyl Laboratory, A301-583A), anti-BRD3 (Bethyl Laboratory, A301-368A), anti-HNF4A (Abcam, ab41898), anti-GR (Thermo Scientific, PA1-511A), anti-STAT3 (Santa Cruz, sc-482) and anti-STAT5 (Santa Cruz, sc-835)
Peak calling parameters	MACS2 callpeak using broad peakcalling with a broad-cutoff of 0.1 for Sham H3K27ac, Sham H3K4me3 and IRI H3K4me3; MACS2 callpeak using broad peakcalling with a broad-cutoff of 0.05 for IRI H3K27ac
Data quality	fastqc reports were done for each sample;
Software	fastqc (v 0.11.6); trimmomatic (v 0.36); bowtie (v 1.1.2); samtools (v 1.3.1); homer (v 4.8.2); MACS2 (v 2.1.1);<br>BEDtools (v 2.26.0); ROSE (super-enhancer) algorithm; GREAT;
